# A Devastating Tear: Atypical Presentation of Aortic Dissection Discovered on Echocardiography

**DOI:** 10.7759/cureus.39757

**Published:** 2023-05-31

**Authors:** Saleh Alhalaseh, Salomon Chamay, Nelson Barrera, Lynn Zaremski, Salim Baghdadi

**Affiliations:** 1 Internal Medicine, SBH (St. Barnabas Hospital) Health System, Bronx, USA; 2 Medicine/Cardiology, SBH (St. Barnabas Hospital) Health System, Bronx, USA; 3 Medicine/Electrophysiology, SBH (St. Barnabas Hospital) Health System, Bronx, USA

**Keywords:** atypical presentation, pocus, echocardiography, hypotension, type b aortic dissection, aortic diseases, type a aortic dissection

## Abstract

Acute aortic dissection (AAD) is a serious medical problem that requires prompt recognition in order to prevent deadly complications. Nevertheless, making the diagnosis can often be challenging. The clinical signs and symptoms of AAD may vary depending on the location of the dissection, leading to subtle differences in the initial patient presentation. Moreover, the classically described signs of blood pressure disparity, pulse deficit, or the presence of a diastolic murmur are often absent. Here, we report a challenging case of AAD in which the patient presented with acute substernal chest pain that resolved after a short period and was associated with hypotension. His bilateral upper and lower extremities were well perfused with symmetrical, palpable pulses. The initial point-of-care ultrasound (POCUS) showed a small pericardial effusion, and a follow-up echocardiogram revealed an ascending aortic flap with aortic root dilation diagnostic of AAD. Our aim is to shed light on the challenge of diagnosing AAD.

## Introduction

Acute aortic dissection (AAD) occurs as a result of a propagating tear within the aortic intima leading to the separation of the vessel intima from the adventitia [1]. The incidence of dissection of the thoracic aorta is estimated to be three to four cases per 100,000 persons per year [2]. However, the reported incidence rate may be underestimated secondary to underdiagnosis. Additionally, cases appear to be increasing, likely due to greater sensitivity and improvement of diagnostic imaging modalities. Rupture of the aorta is the main cause of death in cases of dissection. Risk factors for aortic dissection include uncontrolled chronic hypertension, older age, male sex, with a male-to-female ratio of 3:1, and congenital disorders [1]. Chest pain is the most common symptom, is present in 90% of patients, and is classically described as tearing or ripping in nature [1]. Other clinical findings include hypertension, pulse deficits, diastolic murmur, and focal neurological deficits [1,3].

Chest X-ray (CXR) may demonstrate mediastinal or aortic notch widening. However, the sensitivity and specificity of CXR is low. Computed tomographic angiography (CTA) is considered the gold standard diagnostic imaging modality and may aid in determining the extent of dissection for surgical planning. CTA is readily available and counts with a sensitivity of 98-100%. Transesophageal echocardiogram (TEE) and magnetic resonance imaging (MRI) are additional sensitive tests in the diagnosis of AAD. On the other hand, a two-dimensional (2D) transthoracic echocardiogram (TTE) has a reported sensitivity of 77-80% and a specificity of 93-96% for proximal AAD, with a lower sensitivity for distal aortic dissection [4]. 

The management of AAD depends mainly on the type of dissection according to the Stanford classification. Type A dissections involve the ascending aorta, while type B dissections are those located within the descending aorta. Surgery is the recommended approach in type A dissections, with a reported mortality rate of 18% and an increase in mortality of 1-2% for every hour of delay until surgery [4]. Cases of type A dissection managed medically reach a mortality of 57%. In contrast, type B dissections possess an overall in-hospital mortality of 12%, and cases managed with open surgery vs. medical therapy have a mortality rate of 30% vs 10%, respectively [5]. 

We present a challenging case of extensive aortic dissection with the absence of classically described signs, subtle findings on initial bedside point-of-care ultrasound (POCUS), and subsequent discovery on echocardiography.

## Case presentation

A 64-year-old male with a past medical history of hypertension, hyperlipidemia, prediabetes, smoking (quit smoking more than 20 years ago), and remote history of cocaine use was brought by emergency medical services (EMS) complaining of acute onset chest pain at rest. The chest pain began 30 minutes prior to the emergency department (ED) arrival. The pain was described as substernal in location, pressure-like in quality, 10/10 in intensity, with radiation towards his neck, and associated with nausea and dizziness. The patient reported taking an over-the-counter medication for sexual enhancement purchased at a bodega and shortly afterward developed symptoms while watching television. Prior to arrival, the patient was hypertensive with a blood pressure of 160/80 mmHg reported by EMS. He was given aspirin 324 mg en route to the ED. In the ED, he was noted to be hypotensive at 84/51 mmHg (measured in the right upper arm while supine), with improvement after intravenous (IV) fluid administration. Other vital signs were: heart rate of 62 beats per minute, temperature 98.8 F (37.1 C), and O2 saturation of 97% on room air.

On initial physical examination, the patient was comfortable, awake, and alert. Heart sounds were normal with S1, S2, and regular rate and rhythm. Lungs were clear to auscultation bilaterally. The abdomen was soft and non-tender to palpation. Extremities were warm with palpable, symmetrical pulses throughout all four extremities. 

**Figure 1:**
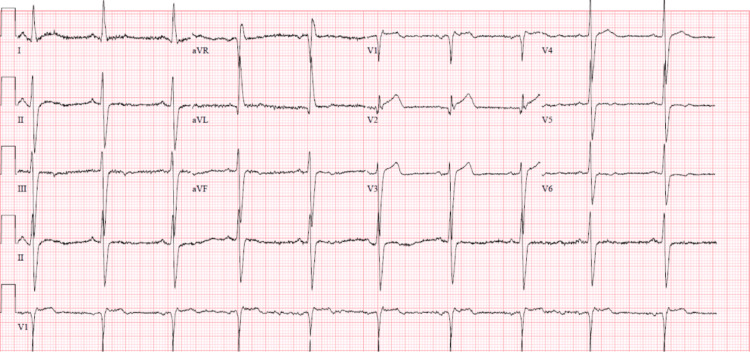
First electrocardiogram obtained demonstrating normal sinus rhythm with ventricular rate of 60 BPM; left axis deviation and LVH criteria also present. LVH: left ventricular hypertrophy, BPM: beats per minute

**Figure 2:**
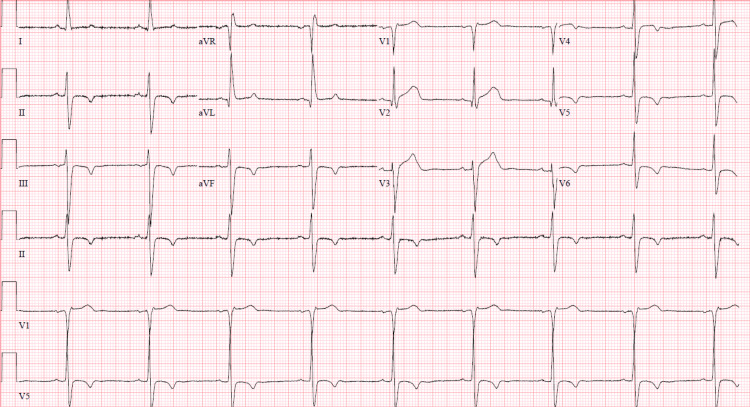
Subsequent electrocardiogram obtained five hours after initial presentation demonstrating sinus bradycardia and a more pronounced T wave inversion in lead II, III, aVF, V4-V6.

An initial electrocardiogram (ECG) in the ED revealed a normal sinus rhythm with <0.5 mm elevation in V1 and LVH, similar to an ECG found in prior records (Figure 1). Serum troponin I was obtained to rule out acute coronary syndrome with negative values on arrival, at four hours, and at seven hours. A repeat ECG five hours after the initial presentation showed sinus bradycardia with T-wave inversions in the inferior and inferolateral leads (Figure 2). CXR reported mild cardiomegaly and pulmonary congestion. 

At the time of the repeat ECG, the patient denied chest pain and instead complained of epigastric pain with two episodes of non-bloody emesis and two episodes of diarrhea mixed with bright red blood. Blood pressure was 110/70 mmHg in the left arm without a major difference in the opposite extremity. He was awake, alert, and oriented without focal neurological deficits. Due to concern for dynamic ECG changes in a patient with high cardiovascular risk for coronary artery disease, the patient was transferred to the telemetry unit for further monitoring. Two hours later, the patient was noted to be somnolent, complaining only of epigastric pain, nausea, and non-bilious, non-bloody vomiting. At this point, blood pressure on the right arm was 96/53 mmHg, with a similar measurement on the contralateral arm. Additionally, the patient was tachycardic at 125 beats per minute, and his pulses were full and equal bilaterally.

Laboratory studies are shown in Table 1. A urine drug screen was requested but was not done due to the emergent nature of the case.

**Table 1:** Laboratory test results with reference ranges WBC: white blood cell count, BUN: blood urea nitrogen, ALT: alanine aminotransferase, AST: aspartate aminotransferase, BNP: brain natriuretic peptide

Laboratory Test	Value	Normal Value
Hemoglobin	13.2 g/dL	12-16 g/dL
WBC	11.3 x 10^3/µL	4.8-10.8 x 10^3/µL
Sodium	140 mEq/L	135-145 mEq/L
Potassium	4.4 mEq/L	3.3-5.3 mEq/L
Chloride	101 mEq/L	96-108 mEq/L
Bicarbonate	23 mg/dL	23-30 mg/dL
Creatinine	1.6 mg/dL	0.6-1.2 mg/dL
BUN	23 mg/dL	8-23mg/dL
Albumin	3.6 mg/dL	3.8-5.0 mg/dL
Total bilirubin	0.4 mg/dL	0.1-1.2 mg/dL
ALT	13 IU/L	4-36 IU/L
AST	18 IU/L	8-33 IU/L
BNP	24 pg/ml	<100 pg/ml

**Figure 3:**
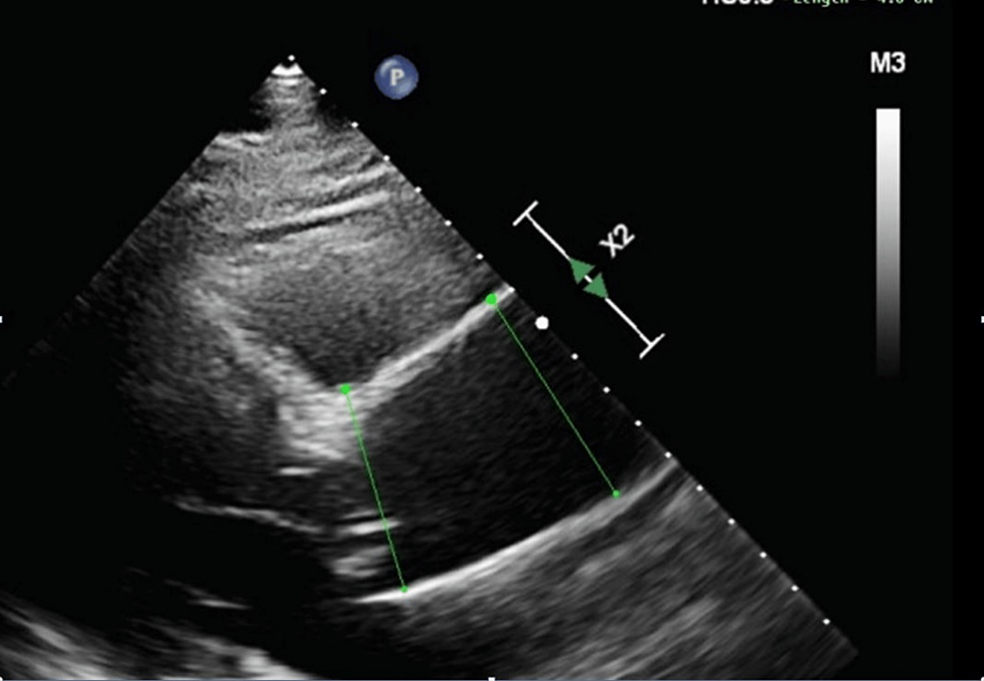
Echocardiogram; dilated ascending aorta

**Figure 4:**
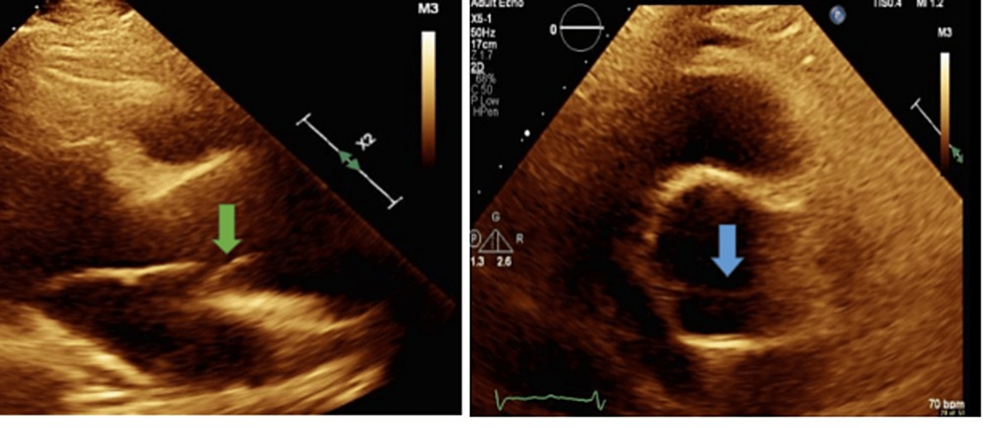
Left: Aortic flap noted on parasternal long axis view (green arrow). Right: Parasternal short view on echocardiogram (blue arrow)

**Figure 5:**
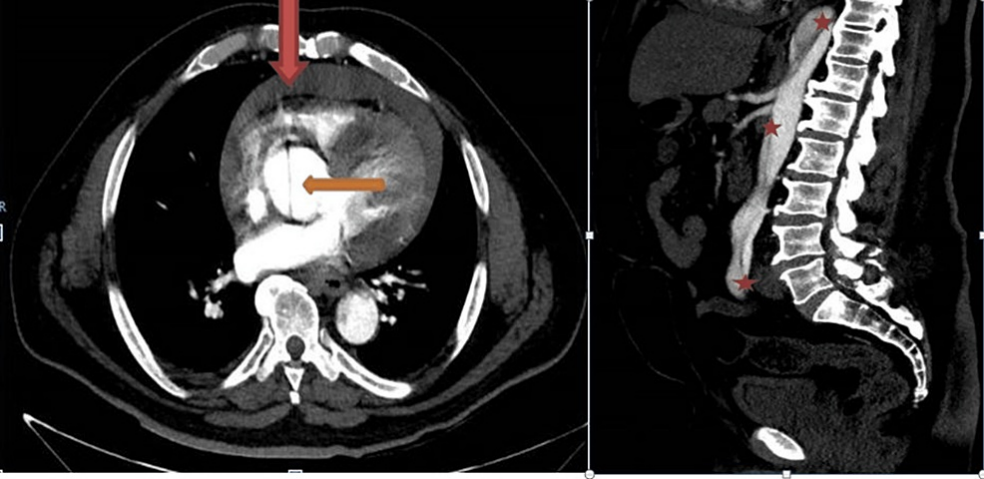
Left: Axial view of computed tomography; red arrow showing hemopericardium; Orange arrow indicating dissecting aorta. Right: Sagittal computed tomography section; three red stars mark the dissection line.

A TTE was done approximately seven hours after the initial presentation and was reported by the reading cardiologist as aortic root dilation, moderate aortic regurgitation, possible flap in the proximal aorta, and small pericardial effusion (Figures 3, 4). A close tertiary center was contacted immediately for transfer and emergent surgical intervention. At the same time, a CTA of the chest, abdomen, and pelvis was obtained emergently and showed a ruptured type A dissection with moderate bloody pericardial effusion. Additionally, the dissecting tear demonstrated extension into all of the aortic root branches down to the bilateral iliac arteries without malperfusion of extremities (Figure 5). The patient's migratory pain and gastrointestinal symptoms were attributed to gastric and mesenteric involvement of the aortic dissection. The patient was transferred for emergent cardiothoracic surgery. At the tertiary center, the patient was noted to not be in acute distress; the cardiac examination was remarkable for tachycardia, the abdominal examination was unremarkable, and extremities were warm and well perfused with 2+ radial and posterior tibialis pulses bilaterally. He was taken to surgery but had a cardiac arrest intra-operatively with cardiac tamponade from a free rupture of the posterior aortic root. A salvage operation was attempted by utilizing a bypass and performing an aortic root and hemiarch replacement. Due to profound hypotension, a decision was made to place the patient on extracorporeal membrane oxygenation (ECMO). However, the patient failed to regain mental status and died of complications from ECMO on the fourth postoperative day.

## Discussion

According to multiple reports, aortic dissection is a frequently misdiagnosed medical entity. A systematic review done by Lovatt et al. found that one out of three (33.8%) patients with AAD was initially misdiagnosed [6], while another retrospective study reported an initial misdiagnosis in 31% of patients [7]. Acute coronary syndrome was the most frequent misdiagnosis in these cases, which can lead clinicians to complicate the course of the disease further by administering anti-platelet therapy and heparin. In our case, the patient's initial diagnostic impression was angina with concern for coronary artery disease, given his risk factors and history of cocaine use. 

Classically, AAD presents with abrupt chest pain described as “tearing” or “sharp,” and can radiate to the neck in type A and to the back in type B [8]. If the dissection propagates to the carotid artery, neurologic symptoms may be present. Symptoms of limb or visceral ischemia can develop if extension into the abdomen occurs. Standard physical examination of the patient includes measurement of blood pressure in both arms, palpation of upper and lower extremity pulses, and cardiac auscultation. Findings indicative of AAD include blood pressure and pulse discrepancy between extremities and diastolic murmur suggestive of aortic insufficiency. 

According to the International Registry of Aortic Dissection (IRAD), physical examination findings suggestive of AAD are often absent [5]. In a retrospective study looking at IRAD from 1996 to 2001, Tsai et al. found that 29% of patients with AAD had hypotension, and only 13% presented with hypotension on arrival [9]. Hypotension was associated more frequently with type A dissections, individuals older than 70 years of age, female sex, neurological deficits, syncope, pulse deficits, and altered mental status. Furthermore, 50% of patients who presented with hypotension died in the hospital compared to only 10% of those without hypotension.

In a separate study, Evangelista et al. analyzed 20 years of IRAD database information and found hypotension at the time of presentation in approximately 25% of patients [5]. The registry found that sudden onset severe chest pain was the most frequent symptom occurring in 79% of patients with type A dissection vs. 63% with type B dissection. Tearing, ripping, or migratory pain were uncommon descriptors. No pain or other neurological findings was recorded in 2.2%, while migratory pain was reported in 16% of patients [10]. Only 40% of type A dissections presented with an aortic insufficiency murmur. Additionally, pulse deficits were detected in 30% of type A and 20% of type B AAD. Evangelista et al. concluded that at hospital presentation, one of the most ominous findings on physical examination was hypotension.

Multiple risk factors have been associated with the development of AAD including age (with a mean age of 63), smoking history, male sex, and pre-existing thoracic aneurysm or genetic syndromes such as Marfan Syndrome. In our case, the patient had a history of hypertension and a remote history of smoking more than 20 years ago. His initial presentation, however, made the diagnosis difficult as he presented with hypotension, along with a lack of pulse deficits or appreciable murmurs.

An interesting aspect of our case was our patient’s history of taking an unknown sexual enhancement drug purchased at a local bodega, just 30 minutes prior to experiencing symptoms. In the literature, Chiang et al. described the potential dangers of counterfeit phosphodiesterase inhibitors (PDE-5i) [11]. It was found that active sildenafil concentration from counterfeits ranged from 0% to 200% of indicated strength. Additionally, many seized counterfeit PDE-5i analyzed in laboratories contained contaminants, among which amphetamines were an implicated contaminating substance. In our case, we were unable to find the exact drug taken by the patient or its composition. However, there may have been a relationship between the drug taken and the patient’s atypical presentation of hypotension. An experimental study published in the Journal of the American Heart Association concluded that PDE-5i may aggravate an existing aortic aneurysm and can contribute to AAD [12]. 

Recently, POCUS has been used with increased frequency at the bedside, especially in emergency departments as it is non-invasive and readily available. Common findings of AAD on POCUS include an intimal aortic flap, aortic root dilation, valve dilation, aortic regurgitation, regional wall motion abnormalities likely due to occlusion of coronary arteries from the dissection, and pericardial effusion, which should raise suspicion for hemopericardium. Importantly, however, the absence of these findings does not rule out a diagnosis of AAD. Our patient was noted to have a small pericardial effusion on POCUS. Therefore, knowledge of specific POCUS findings is paramount for prompt recognition and action in cases of ascending AAD. 

Aside from sudden onset chest pain, additional symptoms of classic AAD were absent in our patient’s case. His initial pressure-like chest pain resolved within the first hour and progressed to gastrointestinal symptoms, likely brought on by the dissection’s migratory nature and involvement of gastric and mesenteric branches of the aorta. Migratory pain is a rare finding of AAD and is reported in only 16% of patients. Additionally, he presented with hypotension on arrival, a finding present in only 13-25% of patients and associated with a poor prognosis. Lastly, the presence of palpable, symmetrical pulses in all four extremities with adequate perfusion and lack of appreciable murmur in our patient made it more challenging. AAD should be considered and a high clinical index of suspicion should be maintained in patients that present with acute chest pain, even in the absence of classically described signs and symptoms. Before beginning antiplatelet therapy, patients with high-risk chest discomfort should be assessed for AAD. Knowledge of specific findings on POCUS, such as aortic root dilation, identification of an aortic flap, and presence of pericardial effusion may aid with prompt recognition of AAD and emergent treatment. 

## Conclusions

This case serves to highlight the importance of maintaining a high clinical index of suspicion for the diagnosis of aortic dissection. The classically described signs and symptoms of AAD, such as tearing or ripping chest pain, pulse deficits, blood pressure discrepancy, and aortic regurgitation murmur, may be absent, even in cases with extensive disease. Although it is a second-line diagnostic modality for aortic dissection, TTE may aid in the detection of ascending aortic dissection as it is non-invasive, readily available, and may prompt urgent intervention. When performing POCUS, searching for aortic root dilation and the presence of pericardial effusion should raise suspicion for ascending aortic dissection.
